# Temporal Cardiorenal Dynamics and Mortality Prediction After TAVR: The Prognostic Value of the 48–72 h BUN/EF Ratio

**DOI:** 10.3390/jcm15020676

**Published:** 2026-01-14

**Authors:** Aykan Çelik, Tuncay Kırış, Fatma Kayaaltı Esin, Semih Babacan, Harun Erdem, Mustafa Karaca

**Affiliations:** Department of Cardiology, İzmir Atatürk Training and Research Hospital, İzmir Katip Çelebi University, 35620 İzmir, Türkiyesemih.babacan.99@gmail.com (S.B.); drharunerdem@gmail.com (H.E.);

**Keywords:** blood urea nitrogen, cardiorenal syndrome, ejection fraction, mortality, transcatheter aortic valve replacement

## Abstract

**Background:** Renal and cardiac dysfunction are major determinants of adverse outcomes following transcatheter aortic valve replacement (TAVR). The ratio of blood urea nitrogen to left ventricular ejection fraction (BUN/EF) integrates renal and cardiac status into a single physiological index. This study aimed to evaluate the prognostic value of both baseline and temporal (48–72 h) BUN/EF ratios for predicting mortality after TAVR. **Methods:** A total of 429 patients (mean age 76 ± 8 years; 51% female) who underwent TAVR for severe aortic stenosis between 2017 and 2025 were retrospectively analyzed. The primary endpoint was long-term all-cause mortality; in-hospital mortality was secondary. Receiver operating characteristic (ROC) curves, Cox regression, and reclassification metrics (NRI, IDI) assessed prognostic performance. Restricted cubic spline (RCS) analysis explored non-linear associations. **Results:** During a median follow-up of 733 days, overall and in-hospital mortality rates were 37.8% and 7.9%, respectively. Both baseline and 48–72 h BUN/EF ratios were independently associated with mortality (HR = 3.46 and 3.79 per 1 SD increase; both *p* < 0.001). The temporal ratio showed superior discrimination for in-hospital mortality (AUC = 0.826 vs. 0.743, *p* = 0.007). Adding baseline BUN/EF to EuroSCORE II significantly improved model performance (AUC 0.712 vs. 0.668, *p* = 0.031; NRI = 0.33; IDI = 0.067). RCS analysis revealed a linear relationship for baseline and a steep, non-linear association for temporal ratios with mortality risk. **Conclusions:** The 48–72 h BUN/EF ratio is a robust dynamic biomarker that predicts early mortality after TAVR, while baseline BUN/EF identifies patients at long-term risk. Integrating this simple bedside index into risk algorithms may refine postoperative monitoring and improve outcome prediction in TAVR populations.

## 1. Introduction

Severe aortic stenosis (AS) is one of the most common valvular diseases in the elderly population. If left untreated, it can cause high mortality and morbidity [1,2]. Transcatheter aortic valve implantation (TAVI), a minimally invasive procedure developed in recent years, has become an effective treatment modality, particularly for patients with high surgical risk. Compared with conservative management, TAVI has been shown to significantly reduce mortality in patients with severe symptomatic aortic stenosis, particularly among elderly individuals with multiple comorbidities [3].

Nevertheless, despite procedural success, post-TAVI mortality remains considerable, reflecting the advanced age, frailty, and high burden of comorbidities characteristic of this population. Knowing the factors affecting short- and long-term mortality after TAVI is important for patient selection and follow-up strategies [4]. In addition to classical risk scores, the importance of simple and routine blood biochemistry parameters has gained increasing importance in recent years [5,6]. In this context, the potential of the blood urea nitrogen (BUN) to ejection fraction (EF) ratio (BUN/EF)—combining BUN as a marker of renal function and hemodynamic instability with EF as an indicator of cardiac function—in predicting mortality has become a new area of research. It was first used by Kiris et al. in 2019 to demonstrate contrast-induced nephropathy in patients with acute coronary syndrome (ACS) undergoing primary percutaneous coronary intervention (PCI) [7]. The BUN/EF ratio is not a newly developed index; rather, it has previously been applied in acute coronary syndromes and chronic coronary artery disease. However, its prognostic significance in patients undergoing TAVI—who exhibit distinct pathophysiological characteristics compared with acute coronary or heart failure cohorts—and particularly its early post-procedural temporal dynamics have not been previously investigated. The present study addresses this gap.

BUN is evaluated as an indirect indicator of cardiovascular load and neurohormonal activation in addition to renal function, and it has been shown to be an independent determinant in acute heart failure (HF) and acute myocardial infarction in studies [8,9]. EF is one of the fundamental indicators of cardiac function, and low EF is a classic indicator of poor prognosis. By reflecting both renal function and left ventricular function, the BUN/EF ratio has allowed for its prediction as a holistic biomarker of mortality risk in a broad perspective ranging from ACS to stable coronary artery disease [8,10].

The TAVI population possesses pathophysiological mechanisms distinct from those of ACS patients. Therefore, the strength and independence of this relationship have not yet been fully established in patient cohorts undergoing TAVI due to severe AS. Unlike previous studies limited to acute coronary or heart failure populations, our study uniquely demonstrates the dynamic prognostic utility of the BUN/EF ratio in the TAVI setting, integrating both baseline and temporal trajectories. In this context, the aim of our current study is to investigate the prognostic value of the BUN/EF ratio on short- and long-term mortality in patients undergoing TAVI for severe AS. We hypothesized that a higher BUN/EF ratio would be associated with increased mortality due to the combined burden of renal dysfunction and reduced cardiac output. The findings obtained may contribute to clinical practice by assisting in patient follow-up and process management with a simple composite physiological index integrating renal biochemical status and cardiac systolic function.

## 2. Materials and Methods

### 2.1. Study Design, Population, and Data Collection

This single-center, retrospective, observational cohort study was conducted at the Department of Cardiology, Izmir Atatürk Training and Research Hospital. A total of 429 consecutive patients who underwent transcatheter aortic valve implantation (TAVI) for severe aortic stenosis between January 2017 and April 2025 were retrospectively analyzed. The patients were divided into two groups based on mortality; survived (*n* = 267), died (*n* = 162). The primary objective was to assess the prognostic value of the BUN/EF ratio.

The study protocol received approval from the Izmir Katip Celebi University, Ataturk Training and Research Hospital ethics committee (approval number: 0684, date: 6 November 2025) and was conducted in accordance with the Declaration of Helsinki. Patient data were extracted from electronic health records via the hospital’s information management system.

#### Inclusion and Exclusion Criteria

Inclusion criteria included:

- Severe aortic stenosis treated with TAVI procedure
- Availability of preprocedural blood urea nitrogen and ejection fraction
- Exclusion criteria included:
- Presence of active malignancy expecting to shorten life expectancy <1-year (with oncological evaluation), active infection, autoimmune disease, or chronic inflammatory conditions
- Missing or incomplete preprocedural laboratory data

Collected variables included demographics, comorbidities, baseline laboratory findings, procedural characteristics, and follow-up outcomes. Severe aortic stenosis was defined as an aortic valve area (AVA) < 1.0 cm^2^, mean transvalvular gradient > 40 mmHg, or peak aortic jet velocity > 4.0 m/s, based on guideline-directed echocardiographic criteria [11]. Blood samples were taken before TAVI. Both echocardiography and BUN measurements were performed on the same day before TAVI. The LVEF was calculated after measuring the end-diastolic and end-systolic left ventricular (LV) volumes in the apical four-chamber and two-chamber views using the modified Simpson’s method. The GFR was calculated with CKD-EPI 2021 formula [12]. Temporal (48–72 h) BUN/EF ratio was calculated by 48–72 h BUN divided by basal LVEF.

### 2.2. Study Endpoint

The primary endpoint of the study was all-cause mortality. Secondary endpoints included in-hospital mortality (death occurring during the index hospitalization after TAVI). The prognostic performance of both baseline and 48–72 h BUN/EF ratios for these outcomes was evaluated.

### 2.3. Statistical Analysis

All statistical analyses were performed using SPSS (Statistical Package for the Social Sciences) version 30.0 (IBM Corp., Armonk, NY, USA) and the R programming language (R Foundation for Statistical Computing, Vienna, Austria, version 4.5.2) within the RStudio environment version 2025.09.2 (Posit PBC, Boston, MA, USA).

Continuous variables were presented as mean ± standard deviation (SD) or median with interquartile range (IQR), depending on the normality of distribution assessed by the Kolmogorov–Smirnov test and visual histograms. Categorical variables were expressed as counts and percentages. Intergroup comparisons were conducted using Student’s *t*-test or the Mann–Whitney U test for continuous variables, and the Chi-square test or Fisher’s exact test for categorical variables, as appropriate.

The predictive performance of the BUN/EF ratio was evaluated using Receiver Operating Characteristic (ROC) curve analysis. The optimal cut-off value was determined using the Youden index (maximum sensitivity + specificity − 1). Comparison of the areas under the curve (AUC) between different models (e.g., BUN/EF ratio vs. individual components) was performed using the DeLong test.

Survival outcomes were estimated using the Kaplan–Meier method, and differences between groups were assessed using the Log-Rank test. Univariate and multivariate Cox proportional hazards regression models were employed to identify independent predictors of mortality and to estimate hazard ratios (HRs) with 95% confidence intervals (CIs). Variables with a *p*-value < 0.10 in the univariate analysis were included in the multivariable models. The assumption of proportional hazards was verified using Schoenfeld residuals.

To further evaluate the prognostic value of the BUN/EF ratio, several advanced statistical methods were utilized:

Reclassification and Discrimination: The incremental predictive value of adding the BUN/EF ratio to the baseline risk model was quantified using the Net Reclassification Improvement (NRI) and Integrated Discrimination Improvement (IDI) statistics.

Restricted Cubic Spline (RCS) Analysis: To assess potential non-linear relationships between the BUN/EF ratio and mortality, RCS analysis was performed with measures of non-linearity (using 3 to 5 knots at fixed percentiles), adjusting for potential confounders.

## 3. Results

### 3.1. Patient Characteristics and Laboratory Findings

The overall mortality rate was 37.8% and in-hospital mortality rate was 7.9%, while median follow-up time was 733 (IQR 288–1192) days. The mean age was 76 ± 8 years, and 51% was female. Non-survived patients were significantly older than surviving patients (78.33 ± 8.2 vs. 75.19 ± 7.69 years; *p* < 0.001). The prevalence of diabetes mellitus (DM), hypertension (HT), dyslipidemia, carotid artery disease, smoking, and coronary artery disease was similar between the groups (*p* > 0.05). However, chronic kidney disease (CKD) was more prevalent in the non-survived group (20% vs. 36%; *p* < 0.001). Non-survived patients had significantly lower LVEF (50.90 ± 11.92% vs. 55.53 ± 9.76%, *p* < 0.001) and higher EuroSCORE II values (12.79 [9.88–18.61] vs. 10.25 [8.53–12.43], *p* < 0.001). Regarding discharge medical therapy, survivors were more frequently treated with statins and ACE inhibitors/ARBs, whereas diuretic use was more common among non-survivors (Table 1). The findings are summarized in Table 1.

Regarding laboratory parameters, glomerular filtration rates (GFRs) (59.26 ± 21.98 vs. 67.98 ± 17.68 mL/min; *p* < 0.001) and albumin levels (3.31 ± 0.52 vs. 3.52 ± 0.50 g/dL; *p* < 0.001) were significantly lower in the non-survived group. There were no significant differences in other laboratory parameters, including hemoglobin (HGB), platelet count (PLT), and white blood cell count (WBC) (*p* > 0.05). The baseline BUN/EF ratio was higher in non-survivors compared to survivors (0.45 [0.32–0.80] vs. 0.35 [0.26–0.48], *p* < 0.001). Additionally, the 48–72 h BUN/EF ratio was higher in non-survivors compared to surviving patients (0.50 [0.30–0.73] vs. 0.28 [0.23–0.44], *p* < 0.001). The findings are shown in Table 2.

### 3.2. Prediction of Overall Mortality and Multivariable Analysis

**Figure 1:**
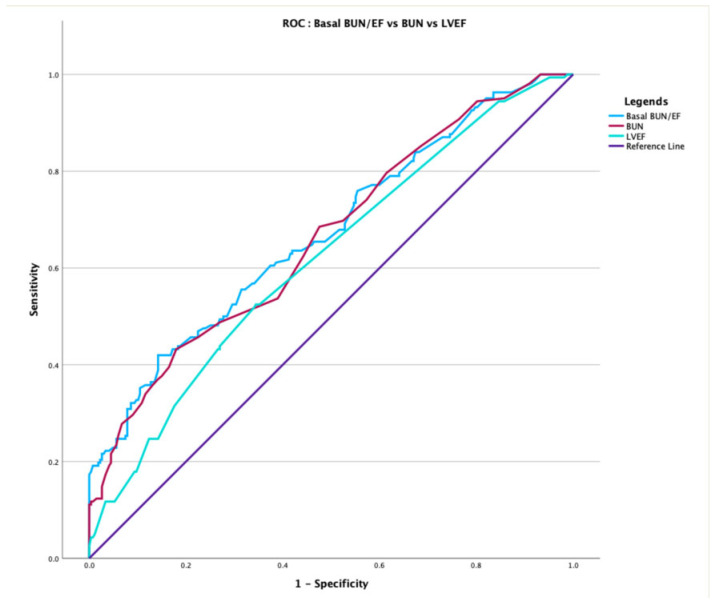
Receiver operating characteristic (ROC) analysis comparing baseline BUN/EF ratio, BUN, and LVEF for long-term mortality prediction. The baseline BUN/EF ratio (red) demonstrated superior discriminative ability (AUC = 0.672, 95% CI: 0.619–0.725, *p* < 0.001) compared with LVEF alone (AUC = 0.619, *p* = 0.043). BUN/EF provided modest improvement over BUN (AUC = 0.662, *p* = 0.397).

For the prediction of all-cause mortality during follow-up, the baseline BUN/EF ratio was identified as an independent predictor in multivariable analysis (HR per 1 SD increase: 3.46, 95% CI: 2.29–5.21; *p* < 0.001, Table 3). The baseline BUN/EF ratio achieved an AUC of 0.672 (95% CI: 0.619–0.725, *p* < 0.001, Figure 1), which was numerically superior to BUN alone (AUC: 0.662, *p* = 0.397) and significantly superior to EF alone (AUC: 0.619, *p* = 0.043). Variables including age, albumin, and BUN/EF ratio with *p* < 0.10 in univariate analyses were entered into the multivariable Cox regression model.

Adding the BUN/EF ratio to a baseline model (Age + Albumin) significantly improved model performance, increasing the AUC to 0.738 (95% CI: 0.681–0.796, *p* = 0.016).

**Figure 2:**
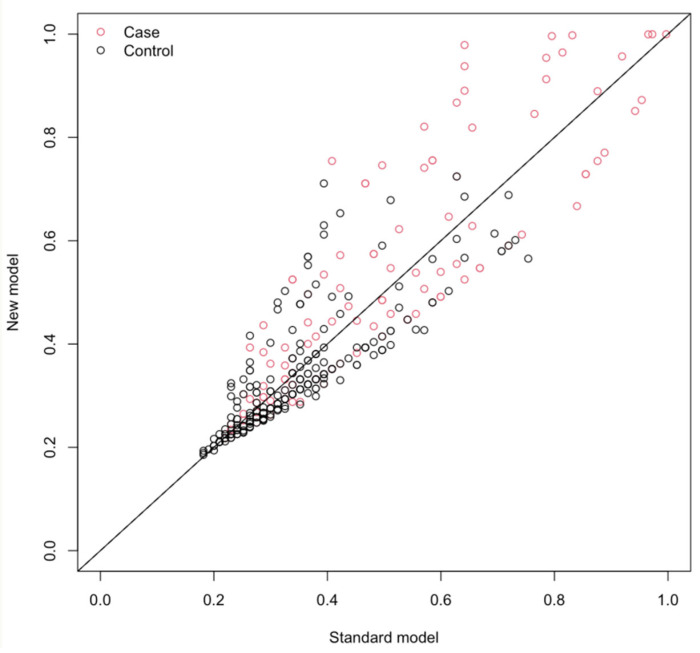
Net reclassification improvement (NRI) scatter plot comparing the baseline model (Age + Albumin) versus the model including BUN/EF ratio. Each point represents an individual’s predicted probability in the standard versus new model. Red circles denote cases (deaths) and black circles denote controls (survivors). Upward shifts indicate improved reclassification of non-survivors with the BUN/EF–augmented model.

Compared with BUN alone, the BUN/EF ratio modestly improved risk reclassification and overall discrimination for mortality. Although the difference in AUCs between the two models was not statistically significant (0.672 vs. 0.662, *p* = 0.397, DeLong test), the BUN/EF ratio achieved a net reclassification improvement (NRI) of 0.21, driven primarily by better identification of non-events (NRI^−^ = 0.37, Figure 2). The integrated discrimination improvement (IDI) was 0.0205 (95% CI 0.0038–0.0379, *p* = 0.018), indicating that the BUN/EF model provided a statistically significant enhancement in the mean separation of predicted probabilities between survivors and non-survivors. The findings are shown in Table 4.

### 3.3. Temporal Dynamics and Prediction of In-Hospital Mortality

To assess the impact of procedural stress on cardiorenal status, we compared the predictive value of baseline versus post-procedural measurements.

**Figure 3:**
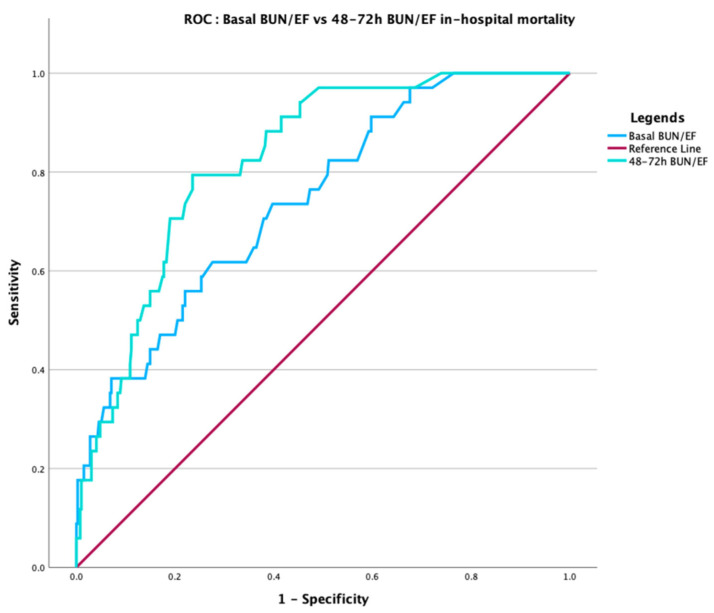
Receiver operating characteristic (ROC) curves comparing baseline and 48–72 h BUN/EF ratios for in-hospital mortality prediction. The temporal (48–72 h) BUN/EF ratio (green) achieved higher discrimination (AUC = 0.826) compared with the baseline ratio (AUC = 0.743; *p* = 0.007, DeLong test), indicating stronger short-term predictive performance after TAVR.

The temporal 48–72 h BUN/EF ratio was a powerful predictor of in-hospital mortality (HR 3.79; 95% CI 2.45–5.86; *p* < 0.001), with an AUC of 0.826, significantly higher than that of the baseline ratio (AUC 0.743; *p* = 0.007, Table 5, Figure 3).

In contrast, for long-term mortality, the baseline BUN/EF ratio showed the strongest independent association (HR 3.61; 95% CI 2.84–4.58; *p* < 0.001), although the temporal ratio still provided a modest improvement in discrimination (AUC 0.720 vs. 0.672; *p* = 0.019, Table 6).

### 3.4. Comparative Performance and Incremental Value over EuroSCORE II

The BUN/EF ratio and EuroSCORE II demonstrated similar predictive performance (AUC = 0.672 vs. 0.668, *p* = 0.898). When compared with EuroSCORE II alone (AUC = 0.668), the addition of the Basal BUN/EF ratio significantly improved the discriminative performance for mortality (AUC = 0.712 vs. 0.668, *p* = 0.031, DeLong test). These findings indicate that while both parameters show comparable individual discrimination, integrating BUN/EF into the EuroSCORE II framework yields a statistically significant incremental prognostic value.

Category-free NRI analysis showed a net reclassification improvement of 0.33 (95% CI 0.15–0.53), driven almost entirely by better reclassification of non-events (NRI^−^ = 0.45, 95% CI 0.34–0.56), while reclassification among events was limited (NRI^+^ = –0.11, 95% CI –0.27–0.04). Consistently, the Integrated Discrimination Improvement (IDI) was 0.067 (95% CI 0.041–0.096, *p* = 2.2 × 10^−6^), indicating a substantial and statistically robust increase in the separation of predicted mortality risk between survivors and non-survivors when BUN/EF was added to EuroSCORE II. The findings are shown in Table 7.

Spearman correlation analysis revealed a moderate positive correlation between the BUN/EF ratio and EuroSCORE II (r = 0.376, *p* < 0.001), indicating that while related, the two indices reflect distinct pathophysiological domains. In a distinct multivariate Cox regression model adjusted for EuroSCORE II, the BUN/EF ratio remained a strong and independent predictor of mortality (HR: 1.92, 95% CI: 1.458–2.549, *p* < 0.001).

### 3.5. Mortality Analysis: Early vs. Late Risk Stratification

**Figure 4:**
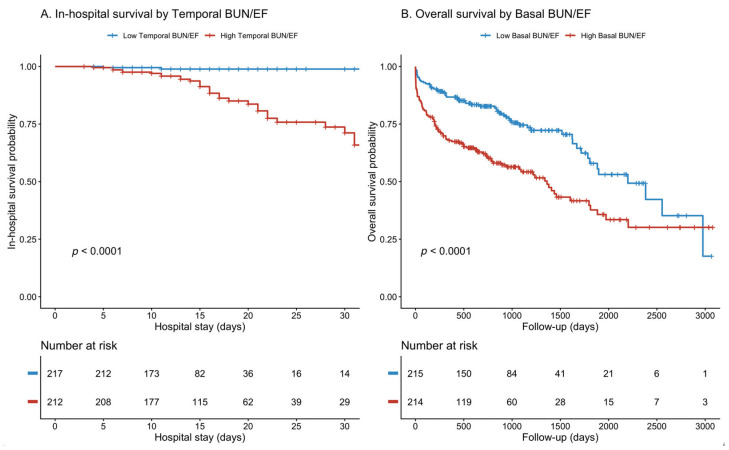
Kaplan–Meier survival analysis stratified by BUN/EF ratio. (**A**) In-hospital survival according to temporal (48–72 h) BUN/EF ratio (log-rank *p* < 0.0001). (**B**) Overall long-term survival according to baseline BUN/EF ratio (log-rank *p* < 0.0001). Patients with higher BUN/EF ratios exhibited significantly worse survival during both early and late follow-up periods. Numbers at risk are shown below each curve. Declining numbers at later time points reflect right censoring due to variable follow-up durations rather than patient survival status.

Kaplan–Meier survival analysis revealed a significant divergence between groups stratified according to the BUN/EF ratio. In the early post-procedural period, patients with a high temporal (48–72 h) BUN/EF ratio had markedly lower in-hospital survival compared with those with a low ratio (log-rank *p* < 0.0001, Figure 4A).

In contrast, during long-term follow-up, the baseline BUN/EF ratio was strongly associated with overall mortality (log-rank *p* < 0.0001, Figure 4B).

### 3.6. Dose–Response Relationship and Clinical Utility

**Figure 5:**
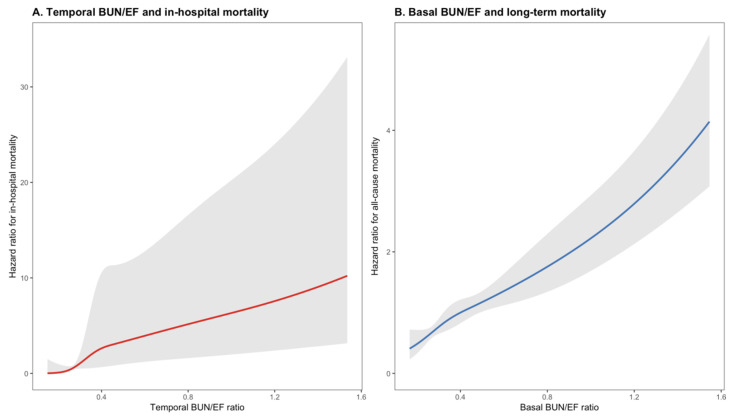
Restricted cubic spline (RCS) curves showing the dose–response relationship between BUN/EF ratio and mortality. (**A**) Temporal (48–72 h) BUN/EF ratio versus in-hospital mortality demonstrated a steep, non-linear association, with risk rising exponentially beyond a ratio of 0.6. (**B**) Baseline BUN/EF ratio versus long-term all-cause mortality displayed an approximately linear relationship, with risk increasing progressively above 0.8. Shaded areas represent 95% confidence intervals.

Restricted cubic spline modeling demonstrated a dose–response relationship between both baseline and temporal BUN/EF ratios and mortality. Restricted cubic spline functions with four knots were applied to flexibly model the association between the BUN/EF ratio and mortality. Knots were placed at the 5th, 35th, 65th, and 95th percentiles of the distribution of each predictor (for baseline BUN/EF: 0.18, 0.32, 0.45, and 1.08; for temporal BUN/EF: 0.17, 0.27, 0.46, and 1.12, respectively). For the baseline BUN/EF ratio, the association with long-term all-cause mortality was nearly linear, showing a steady increase in hazard ratio as the ratio rose (*p* for overall association <0.001, *p* for nonlinearity = 0.12). The risk began to increase more prominently at BUN/EF values above approximately 0.8, reaching a more than fourfold hazard at the upper end of the distribution. In contrast, the temporal 48–72 h BUN/EF ratio showed a markedly steeper and non-linear pattern in relation to in-hospital mortality. The hazard increased exponentially for ratios exceeding approximately 0.6, suggesting an early and acute prognostic impact of cardiorenal stress reflected by the postoperative BUN/EF elevation (Figure 5A,B illustrates these relationships).

## 4. Discussion

To our knowledge, this is the first study to demonstrate an independent association between the high BUN/EF ratio and cardiovascular outcomes in patients with severe aortic stenosis undergoing TAVI. Additionally, this study provides new evidence that early temporal dynamics of the BUN/EF ratio serve as an independent prognostic indicator after TAVR.

The strong association between elevated BUN/EF ratios and adverse outcomes suggests that deaths were likely driven by cardiovascular and cardiorenal mechanisms. Elevated BUN reflects neurohormonal activation, systemic congestion, and impaired renal perfusion, while reduced ejection fraction indicates limited myocardial reserve. In elderly TAVI patients, this combination may predispose to fatal complications such as heart failure decompensation, cardiogenic shock, arrhythmias, or vulnerability to non-cardiac stressors including infection or sepsis.

While prior research has linked preprocedural BUN or EF to adverse outcomes, our data demonstrate that the evolution of this ratio within 48–72 h post-procedure carries even greater predictive value. The 48–72 h BUN/EF ratio exhibited excellent discrimination for in-hospital mortality and retained significance for long-term mortality in multivariable models.

From a clinical perspective, routine calculation of the BUN/EF ratio and its 48–72 h evolution may provide an inexpensive, bedside method for identifying high-risk patients during the vulnerable early post-TAVR phase. The concept of dynamic cardiorenal monitoring offers a pragmatic addition to established surgical risk scores, particularly in elderly patients or those with preserved ejection fraction, in whom subtle renal–myocardial imbalance often precedes overt decompensation. Implementing this simple ratio in daily practice could facilitate timely optimization of diuretic therapy, hemodynamic support, and follow-up intensity. Future multicenter and prospective studies should validate these findings and determine whether incorporating BUN/EF ratio into post-TAVR risk algorithms improves clinical decision-making and outcomes.

Renal dysfunction is known to be associated with severe AS [13]. Chronic kidney disease can accelerate AS through pathways such as mineral metabolism disorders, inflammation, oxidative stress, and hemodynamic overload. It has been shown that even small changes in GFR in early-stage CKD patients can increase the progression of aortic valve calcification in patients at high risk for coronary atherosclerosis [14]. High urea levels can be evaluated as a burden (neurohumoral overload) indicating reduced GFR, increased urea production, or hemodynamic disturbances, which increases the risk of complications after TAVI. Excessive activation of the renin–angiotensin–aldosterone system, sympathetic nervous system, and arginine vasopressin system points to both renal dysfunction and progression of heart failure [15].

The relationship between renal dysfunction and mortality in TAVI patients is well known in the literature. In a study by Sokmen et al., it was shown that BUN, creatinine levels, and the degree of chronic kidney disease were associated with post-TAVI mortality [16]. In another study, serum urea levels and GFR were shown to be independent predictors of post-TAVI mortality [17].

Ejection fraction is one of the fundamental indicators of cardiac function. However, studies have shown that low EF alone is insufficient to predict mortality in TAVI patients [18]. Some studies investigating EF changes in patients after the TAVI procedure have shown that recovery of EF has positive effects on mortality and patient outcomes [19,20].

With the BUN/EF ratio, the combined evaluation of both the hemodynamic/neurohumoral status and the myocardial reserve of the patients gathers these mechanisms under a single roof. The relationship between a high BUN/EF ratio and mortality in TAVI patients may be due to the following mechanisms. In the post-TAVI period, the withdrawal of perioperative inotropic support while myocardial recovery remains minimal causes additional stress and can trigger cardiogenic shock [21]. Elevated BUN is a sign of electrolyte imbalance, and its combination with depressed EF can lead to fatal arrhythmias. Patients who still have depressed EF and high BUN despite TAVI may be resistant to medical treatment. Recurrent heart failure hospitalization is an independent predictor of prognosis [22,23]. In patients with renal dysfunction and low myocardial reserve, conditions such as sepsis and pneumonia can rapidly evolve into cardiogenic shock [21].

### 4.1. Pathophysiological Rationale

The present study demonstrated that both baseline and temporal BUN/EF ratios are independently associated with adverse outcomes following TAVI, reflecting distinct phases of cardiorenal interaction. The BUN/EF ratio integrates two critical physiological axes—renal perfusion and left ventricular systolic function—capturing the combined effects of forward failure and renal metabolic stress.

An elevated baseline BUN/EF ratio likely reflects chronic cardiorenal maladaptation, characterized by sustained neurohormonal activation, impaired renal blood flow, and subclinical volume overload. Conversely, a sharp postoperative increase (temporal BUN/EF) may indicate acute renal stress or transient hemodynamic compromise following TAVI, capturing a dynamic deterioration in the early post-procedural phase.

Although the AUC values of BUN and BUN/EF ratio appeared statistically comparable, further analysis revealed the true value of the combined parameter. First, an interaction analysis demonstrated a significant interplay between BUN and LVEF, indicating that the prognostic impact of BUN is significantly modulated by cardiac function. This statistical proof supports the biological rationale that cardiorenal interaction creates a synergistic risk beyond the sum of individual components.

The BUN/EF ratio amplifies subtle changes in both renal and cardiac function thereby unmasking patients with latent cardiorenal imbalance. While a slight decrease in EF or a mild increase in BUN might be overlooked individually, their ratio amplifies these subtle maladaptive changes, revealing a hidden state of hemodynamic stress.

Although we utilized LVEF as the primary cardiac metric, we acknowledge that strain-based echocardiographic parameters, such as Global Longitudinal Strain (GLS), may detect subtle myocardial dysfunction earlier than LVEF. Recent studies have shown that GLS is a sensitive marker for prognosis in aortic stenosis [24,25]. Future research integrating the BUN/GLS ratio could potentially refine risk stratification further.

The BUN component reflects subclinical congestion and neurohumoral activation that EF alone fails to capture. Consequently, an elevated BUN/EF ratio effectively unmasks high-risk phenotypes among patients who might otherwise be classified as low-risk based solely on conventional echocardiographic parameters.

### 4.2. Temporal vs. Baseline Predictive Patterns

TAVI represents a significant hemodynamic challenge, akin to a physiological ‘stress test’ for the cardiorenal axis. While baseline parameters reflect the patient’s chronic homeostatic reserve, the dynamic change (delta) in the BUN/EF ratio at 48–72 h unveils the acute adaptive—or maladaptive—response to procedural stress. A worsening ratio during this critical window signifies an inability to accommodate the rapid hemodynamic shifts caused by rapid pacing, contrast media exposure, and altered cardiac output. Thus, the temporal trajectory of the BUN/EF ratio offers a more granular risk stratification than static baseline measurements alone, capturing the ‘active’ deterioration of cardiorenal coupling”. Our restricted cubic spline analysis revealed divergent dose–response patterns between the early and late phases. Temporal BUN/EF exhibited a steep, non-linear (J-shaped) relationship with in-hospital mortality, whereas the baseline BUN/EF ratio showed a more linear, gradual association with long-term all-cause mortality. These findings suggest that temporal BUN/EF may serve as an acute marker of early decompensation or procedural-related renal injury, while baseline BUN/EF predominantly reflects chronic systemic vulnerability. The non-linear pattern observed for temporal BUN/EF implies that even moderate elevations can disproportionately amplify short-term mortality risk, underlining its potential utility in immediate postoperative monitoring and decision-making.

### 4.3. Comparison with Previous Studies

TAVI candidates generally consist of elderly, frail patients with multiple comorbidities. Models such as STS and EuroSCORE used for this population can sometimes be complex and may not fully reflect the patient’s frailty [26]. Remarkably, a simple ratio derived from routine bedside parameters achieved a discriminative ability statistically comparable to the complex, multi-variable EuroSCORE II. More importantly, the addition of the BUN/EF ratio to EuroSCORE II significantly improved risk prediction accuracy, as evidenced by a Net Reclassification Improvement (NRI) of 34%. This improvement was largely driven by the correct reclassification of survivors (Non-event NRI ~0.50), indicating that incorporating the BUN/EF ratio can effectively reduce ‘false positive’ high-risk labels, allowing clinicians to more accurately identify patients with a favorable trajectory despite a high baseline risk score.

The BUN/EF ratio, on the other hand, is a simple parameter that does not require extra cost and can be easily calculated at the bedside. Rather than replacing complex scores, BUN/EF is an additional parameter for quick bedside risk stratification. It may point to a high-risk group that requires closer follow-up after TAVI and where hydration and diuresis therapy must be managed with precision. Our study contributes to the limited number of analyses in the literature focusing on the combination of renal and ventricular function.

Previous studies have emphasized the prognostic role of isolated BUN, creatinine, or estimated GFR in TAVI populations; however, their predictive performance has been modest. The current analysis extends these observations by demonstrating that integrating BUN with EF—into a single physiologically coherent ratio—enhances discriminatory ability.

Our findings align with the cardiorenal coupling paradigm previously described in acute heart failure cohorts and in chronic systolic dysfunction, yet this is among the first studies to demonstrate its applicability in the context of TAVI [27,28]. Moreover, the strong AUC difference between temporal and baseline ratios (0.83 vs. 0.74 for in-hospital mortality) supports the incremental prognostic utility of dynamic renal–cardiac interplay over static preoperative parameters.

### 4.4. Clinical Implications

Clinically, the BUN/EF ratio represents a simple, readily available marker that could enhance risk stratification after TAVI without requiring additional laboratory or imaging resources. A temporal increase in the ratio within 48–72 h may identify patients at high risk of early postoperative mortality, potentially warranting closer hemodynamic monitoring, volume optimization, or early nephrology consultation.

In contrast, a high baseline BUN/EF ratio may assist in long-term risk assessment and patient counseling, as it identifies individuals with chronic cardiorenal dysfunction who may derive less survival benefit despite procedural success.

Incorporating both baseline and temporal measures into clinical pathways may thus enable phase-specific prognostication—acute vs. chronic—offering a more comprehensive assessment of patient vulnerability.

In summary, the RCS analysis confirms a distinct temporal heterogeneity in the prognostic impact of the BUN/EF ratio. Temporal (post-procedural) elevations carry a disproportionate risk for in-hospital mortality through a non-linear mechanism, while baseline ratios show a linear, sustained relationship with long-term outcomes. These observations underscore the importance of dynamic renal–cardiac evaluation in the continuum of TAVI care.

## 5. Limitations

This study has several limitations. First, it was a single-center retrospective analysis, which may limit the generalizability of the findings. Due to the retrospective nature of the study, cause-specific mortality data were not systematically available, precluding a detailed analysis of cardiovascular versus non-cardiovascular causes of death. Accordingly, the primary endpoint was all-cause mortality, which may limit mechanistic interpretation but provides a robust and unbiased outcome measure in this elderly, high-risk population.

Second, although serial measurements were standardized at 48–72 h, individual variations in timing and postprocedural management could have influenced BUN or EF values. Third, we did not perform external validation in an independent cohort; thus, our findings should be interpreted as hypothesis-generating. Fourth, echocardiographic EF was used as a surrogate for systolic function rather than strain-based parameters, which might provide additional insight into subtle myocardial changes. Additionally, basal LVEF was used when calculating the temporal 48–72 h BUN/EF ratio. Finally, the study did not account for procedural details such as contrast volume or periprocedural hypotension, which may also affect renal function. Despite these limitations, the large sample size, standardized measurement intervals, and advanced statistical validation strengthen the reliability of our conclusions.

## 6. Conclusions

In patients undergoing TAVR, the 48–72-h BUN/EF ratio provides a powerful, easily obtainable dynamic biomarker that independently predicts both in-hospital and long-term mortality. Temporal worsening of this ratio—rather than baseline elevation—identifies a subgroup of patients experiencing maladaptive cardiorenal interaction, particularly among those with preserved ejection fraction.

Most importantly, the BUN/EF ratio provides significant incremental prognostic value over the standard EuroSCORE II, particularly by improving risk stratification among survivors. These findings suggest that integrating this simple cardiorenal index into current risk assessment algorithms could refine outcome prediction and guide personalized management strategies in the TAVI population.

Recognizing these dynamic risk trajectories may enable clinicians to anticipate early decompensation, optimize management, and personalize surveillance intensity after valve implantation. Incorporating simple bedside indices such as the BUN/EF ratio into post-TAVR risk algorithms could refine outcome prediction and improve patient care. Integration of the BUN/EF ratio into prospective multicenter registries may help redefine postoperative risk phenotyping in TAVI patients.

## Figures and Tables

**Table 1:** Demographic and Clinical Characteristics of TAVI Patients by mortality status.

Characteristics (n, %)	Survived (*n* = 267)	Non-Survived (*n* = 162)	*p*-Value
Age (years)	75.19 ± 7.69	78.33 ± 8.2	<0.001
Gender (Male)	129 (48.3%)	83 (51.2%)	0.558
DM	90 (33.7%)	56 (34.6%)	0.855
HT	186 (69.7%)	100 (61.7%)	0.091
Dyslipidemia	99 (37.1%)	47 (29%)	0.087
Carotid Artery Disease	5 (1.9%)	2 (1.2%)	0.610
Smoking	11 (4.1%)	4 (2.5%)	0.367
CAD	217 (81.2%)	135 (83.3%)	0.474
History of Heart Valve Surgery	9 (3.4%)	1 (0.6%)	0.182
PAD	29 (10.9%)	29 (17.9%)	0.039
Heart Failure	68 (25.5%)	46 (28.4%)	0.506
COPD	18 (6.7%)	20 (12.3%)	0.048
Previous Stroke	20 (7.5%)	11 (6.8%)	0.786
CKD n (%)	52 (19.5%)	58 (35.8%)	<0.001
Previous AF	63 (23.6%)	48 (29.6%)	0.167
PCI Before TAVI	23 (8.6%)	20 (12.3%)	0.212
LVEF (%)	55.53 ± 9.76	50.90 ± 11.92	<0.001
EUROSCORE 2 ^†^	10.25 (8.53–12.43)	12.79 (9.88–18.61)	<0.001
Discharge Medications			
Antiplatelet therapy	257 (96.3%)	137 (84.6)	<0.001
VKAs	25 (9.4%)	11 (6.8)	0.351
DOACs	39 (14.6%)	18 (11.1%)	0.301
Beta-blockers	144 (53.9%)	90 (55.6%)	0.743
ACEIs/ARBs	103 (38.6%)	41 (25.3%)	0.005
SGLT2i	8 (3%)	4 (2.5%)	0.748
Diuretics	61 (22.8%)	57 (35.2%)	0.006
MRAs	27 (10.1%)	13 (8%)	0.471
Statins	210 (78.7%)	77 (47.5%)	<0.001

^†^—These values were described by median with inter-quartile range (25th and 75th percentile). Abbreviations: TAVI: Transcatheter Aortic Valve Implantation; CAD: Coronary Artery Disease; DM: Diabetes Mellitus; HT: Hypertension; PAD: Peripheral Artery Disease; COPD: Chronic Obstructive Pulmonary Disease; CKD: Chronic Kidney Disease; LVEF: left ventricular ejection fraction; PCI: Percutaneous Coronary Intervention, ACEIs: Angiotensin-Converting Enzyme Inhibitors; ARBs: Angiotensin Receptor Blockers; VKAs: Vitamin K Antagonists; DOACs: Direct Oral Anticoagulants; SGLT2i: Sodium–Glucose Cotransporter 2 Inhibitors; MRAs: Mineralocorticoid Receptor Antagonists.

**Table 2:** Laboratory Findings in TAVI Patients Based on Mortality.

Parameters	Survived (*n* = 267)	Non-Survived (*n* = 162)	*p*-Value
HGB (g/dL)	11.24 ± 1.72	10.83 ± 2.01	0.023
Lymphocyte (×10^3^/μL) ^†^	1.40 (1.0–1.7)	1.20 (0.89–1.70)	0.027
WBC (×10^3^/μL)	8.05 ± 3.38	8.22 ± 3.18	0.626
PLT (×10^3^/μL)	220.92 ± 81.46	214.38 ± 86.29	0.431
Albumin (g/dL)	3.52 ± 0.50	3.31 ± 0.52	<0.001
GFR (mL/min)	67.98 ± 17.68	59.26 ± 21.98	<0.001
Cre (mg/dL) ^†^	1 (0.85–1.19)	1.12 (0.91–1.44)	<0.001
48–72 h Cre (mg/dL) ^†^	0.98 (0.78–1.19)	1.12 (0.83–1.76)	<0.001
BUN ^†^	19 (15–24)	23 (17–38)	<0.001
48–72 h BUN ^†^	16 (13–22)	24.5 (16–37)	<0.001
CRP (mg/L) ^†^	17 (5.95–55.35)	22 (10–55)	0.398
BUN/EF Ratio ^†^	0.35 (0.26–0.48)	0.45 (0.32–0.80)	<0.001
48–72 h BUN/EF Ratio ^†^	0.28 (0.23–0.44)	0.54 (0.32–0.73)	<0.001

^†^—These values were described by median with inter-quartile range (25th and 75th percentile). HGB: Hemoglobin; PLT: Platelet Count; GFR: Glomerular Filtration Rate; WBC: White Blood Cell Count; Cre: Creatinine, BUN: Blood Urea Nitrogen, CRP: C-reactive Protein, EF: Ejection Fraction.

**Table 3:** Predictors of mortality in univariate and multivariate analysis.

	Univariate			Multivariate		
Variables	HR	95% CI	*p*	HR	95% CI	*p*
Age	1.055	1.027–1.084	<0.001	1.055	1.021–1.091	0.001
Hypertension	0.702	0.466–1.059	0.092			
Dyslipidemia	0.694	0.455–1.056	0.088			
PAD	1.789	1.026–3.123	0.040			
COPD	1.948	0.998–3.805	0.051			
Albumin	0.921	0.881–0.962	<0.001	0.928	0.885–0.974	0.002
Hemoglobin	0.882	0.791–0.984	0.025			
GFR	0.978	0.968–0.988	<0.001			
BUN ^‡^	1.061	1.041–1.083	<0.001			
EF ^‡^	0.962	0.944–0.980	<0.001			
BUN/EF	14.396	6.064–34.172	<0.001	18.328	7.004–47.963	<0.001
BUN/EF ^†^	3.117	2.156–4.505	<0.001	3.455	2.293–5.206	<0.001

^†^ The standardized BUN/EF ratio HR per 1 SD increase. ^‡^ To avoid multicollinearity, BUN/EF was not included in the same multivariable model with its individual components. Separate models were therefore constructed to compare the prognostic performance of the composite index versus its individual parameters. HR: Hazard Ratio; CI: Confidence Interval; GFR: Glomerular Filtration Rate; PAD: Peripheral Artery Disease; COPD: Chronic Obstructive Pulmonary Disease, BUN: Blood Urea Nitrogen; EF: Ejection Fraction.

**Table 4:** Comparison of discrimination and reclassification statistics between BUN and BUN/EF ratio models for mortality.

	AUC	ΔAUC (*p*)	NRI	IDI
BUN (Model 1)	0.662	reference	–	–
BUN/EF (Model 2)	0.672	0.397	+0.210	+0.021 (*p* = 0.018)

AUC: area under the receiver operating characteristic curve; NRI: net reclassification improvement comparing Model 2 with Model 1 (continuous NRI); IDI: integrated discrimination improvement comparing Model 2 with Model 1.

**Table 5:** 48–72 h BUN/EF vs. Basal BUN/EF prediction for in-hospital mortality.

	HR (95% CI)	*p* Value	Concordance	AUC (*p*)
48–72 h BUN/EF	3.79 (2.45–5.86)	<0.001	0.778	0.826 (*p* = 0.007)
Basal BUN/EF	–	–	–	0.743

AUC: area under the receiver operating characteristic curve; HR: Hazard Ratio; CI: Confidence Interval.

**Table 6:** 48–72 h BUN/EF vs. Basal BUN/EF prediction for total mortality.

	HR (95% CI)	*p* Value	Concordance	AUC (*p*)
Basal BUN/EF	3.61 (2.84–4.58)	<0.001	0.674	
48–72 h BUN/EF	–	–	–	0.720 (*p* = 0.019)

AUC: area under the receiver operating characteristic curve; HR: Hazard Ratio; CI: Confidence Interval.

**Table 7:** Incremental prognostic value of Basal BUN/EF ratio beyond EuroSCORE II for predicting in-hospital mortality.

	AUC	ΔAUC (*p*)	NRI	IDI
EuroSCORE II (Model 1)	0.668	reference	–	–
EuroSCORE II + BUN/EF (Model 2)	0.712	0.044	+0.330	+0.067 (*p* < 0.001)

AUC: area under the receiver operating characteristic curve; NRI: net reclassification improvement comparing Model 2 with Model 1 (continuous NRI); IDI: integrated discrimination improvement comparing Model 2 with Model 1.

## Data Availability

The data presented in this study are available on request from the corresponding author due to privacy.
